# Socio-economic predictors of performance in the Undergraduate Medicine and Health Sciences Admission Test (UMAT)

**DOI:** 10.1186/1472-6920-13-155

**Published:** 2013-11-29

**Authors:** Ian B Puddey, Annette Mercer

**Affiliations:** 1 Faculty of Medicine, Dentistry and Health Sciences, University of Western Australia, 35 Stirling Hwy, Crawley, WA 6009, Australia

## Abstract

**Background:**

Entry from secondary school to Australian and New Zealand undergraduate medical schools has since the late 1990’s increasingly relied on the Undergraduate Medicine and Health Sciences Admission Test (UMAT) as one of the selection factors. The UMAT consists of 3 sections – logical reasoning and problem solving (UMAT-1), understanding people (UMAT-2) and non-verbal reasoning (UMAT-3). One of the goals of using this test has been to enhance equity in the selection of students with the anticipation of an increase in the socioeconomic diversity in student cohorts. However there has been limited assessment as to whether UMAT performance itself might be influenced by socioeconomic background.

**Methods:**

Between 2000 and 2012, 158,909 UMAT assessments were completed. From these, 118,085 cases have been identified where an Australian candidate was sitting for the first time during that period. Predictors of the total UMAT score, UMAT-1, UMAT-2 and UMAT-3 scores were entered into regression models and included gender, age, school type, language used at home, deciles for the Index of Relative Socioeconomic Advantage and Disadvantage score, the Accessibility/Remoteness Index of Australia (ARIA), self-identification as being of Aboriginal or Torres Strait Islander origin (ATSI) and current Australian state or territory of abode.

**Results:**

A lower UMAT score was predicted by living in an area of relatively higher social disadvantage and lower social advantage. Other socioeconomic indicators were consistent with this observation with lower scores in those who self-identified as being of ATSI origin and higher scores evident in those from fee-paying independent school backgrounds compared to government schools. Lower scores were seen with increasing age, female gender and speaking any language other than English at home. Divergent effects of rurality were observed, with increased scores for UMAT-1 and UMAT-2, but decreasing UMAT-3 scores with increasing ARIA score. Significant state-based differences largely reflected substantial socio-demographic differences across Australian states and territories.

**Conclusions:**

Better performance by Australian candidates in the UMAT is linked to an increase in socio-economic advantage and reduced disadvantage.This observation provides a firm foundation for selection processes at medical schools in Australia that have incorporated affirmative action pathways to quarantine places for students from areas of socio-economic disadvantage.

## Background

The Undergraduate Medicine and Health Sciences Admission Test (UMAT) has been developed to assist with the selection of students into medicine, dentistry and health science degree programs at an undergraduate level in Australian and New Zealand universities. It comprises 3 subtests which are developed each year by the Australian Council for Educational Research (ACER) on behalf of a group of Australian universities which form the UMAT Consortium [1]. The test is promoted as enhancing a focus on selection based on general attributes and non-academic personal skills gained through prior experience and learning and is designed to complement academic results used in selection processes. In section 1 (UMAT-1) candidates are required to exercise logical reasoning and problem solving skills using both inductive and deductive reasoning with an emphasis on logical argument in working to a solution. The construct and the nature of the items in this section have been consistent over the years. In section 2 (UMAT-2) the emphasis has always been on assessing empathy and emotional intelligence with candidates required to show an understanding of the thoughts, feelings, behaviour and intentions portrayed within each question. Between 2003 and 2004 the section was changed in name from Interaction Skills to Understanding People together with a change in the format of some items. Section 3 (UMAT-3) evaluates a candidate’s non-verbal reasoning skills. It also changed at the same time as Section 2, when the use of ‘embedded figures’ was removed with items subsequently consisting solely of patterns or sequences of shapes. This was consistent with recent literature on the construct and the desire to obtain a measure of cognitive ability which was relatively independent of language ability and specific cultural knowledge.

Changes in selection strategies for admission to medical schools that have incorporated aptitude tests have at least in part been in the belief that they might serve to redress the under representation of students from a lower socio-economic background in medical schools [2-4]. This imbalance has been longstanding and is consistently reported globally [5-7]. It is attributed to a lower number of applicants from those from a more disadvantaged socio-economic background. For example, in the UK those applying to study medicine are more likely to be of higher socio-economic status and from fee paying independent secondary schools and in particular independent schools that exhibit higher levels of academic achievement [8]. As a further example, only 8% of applicants to the 1999 University of Newcastle medical course originated from postcodes linked to those in the lowest socio-economic quartile [9].

In a review of potential approaches to widening access for a broader spread of students across the socio-economic spectrum, Powis et al. [10] recommend the application of tests that measure a range of cognitive skills and non-cognitive personal qualities, with the implication that these tests are diversity neutral. However, with respect to the UMAT we have previously reported that a socio-economic index linked to the secondary school of origin of students entering our medical school predicted higher UMAT scores in those who attended schools with higher socio-economic advantage [5]. We also reported that the introduction of both a structured interview and the score from UMAT had not served to increase socio-economic diversity in secondary school leaver entrants to our medical course [5].

Others who have introduced attribute-based admission criteria as an alternative to grades-based selection in an attempt to increase medical student socio-economic diversity have also failed to see any significant change [11]. These observations question the assumption that simply utilising measures of aptitude as an additional selection tool can help overcome the potential for socio-economic background to influence selection processes that are based entirely on secondary school results or grade point average during tertiary studies. Furthermore, they raise the question as to whether there may be potential socio-economic influences on performance in aptitude tests that may have been previously overlooked. In this respect, recent reports have indicated that socio-economic factors may be determinants of performance in both the Medical College Admission Test (MCAT) [12], widely used for medical student selection in North America, and the UK Clinical Aptitude test (UKCAT), used in medical student selection in the UK since 2006 [13].

An association of a number of demographic variables with overall performance in the UMAT and each of its sections has been regularly observed in annual reports on performance prepared for ACER on behalf of the UMAT Consortium [1] and have also been reported by others [5,14,15]. Generally, males perform better than females in total UMAT score, UMAT-1 and UMAT-3 but less well than females in UMAT-2. Older students underperform relative to their younger counterparts and those from non-English speaking backgrounds perform less well than those from an English speaking background. Those from rural backgrounds perform less well in UMAT-1 and UMAT-3 but not in UMAT-2. Mulivariate linear regression suggests these associations are relatively weak, accounting for only 4.1% of the variance for total UMAT score, 5.4% of the variance in UMAT-1, 11.1% for UMAT-2 and 3.7% for UMAT-3 [1]. However, these annual reports have not considered the potential contribution of socio-economic background in predicting UMAT performance.

We have therefore identified all Australian candidates who sat the UMAT on a first occasion between 2000 and 2012 and linked their postcodes to the Socio-Economic Indexes for Areas generated from the 2006 census data [16]. We have then investigated the associations of relative socio-economic advantage and disadvantage scores with total UMAT score or performance in each of its 3 sections and now report these findings in relation to other already established demographic predictors of UMAT performance. As a further window on any potential socio-economic influences we also report UMAT performance in relation to background secondary school, rural background or self-identification as an Aboriginal or Torres Strait Islander (ATSI).

## Methods

The UMAT results for 158,909 candidates who sat the UMAT between 2000 and 2012 were obtained from ACER. From these, 118,085 cases were identified where an Australian candidate was sitting for the first time during that period. Demographic data collected on enrolment for the UMAT included date of birth, gender, postal address, language spoken at home, type of high school attended and self-identification as being of ATSI origin.

Language spoken at home was classified according to the Australian Standard Classification of Languages (ASCL), 2011 [17]. For multivariate analysis this was collapsed into 4 groups – English, European languages, Asian languages and all Other languages. Type of school was classified into one of 5 groups – government (publicly funded), independent (fee paying), Catholic, Technical and Further Education institutions (TAFE – public provider of predominantly vocational tertiary education courses) and Other.

Socio-economic status was imputed from each candidate’s correspondence postcode at the time of first sitting the UMAT by linking it to the Socio-Economic Indexes for Areas (SEIFA) generated from the 2006 census data [16]. We used the deciles generated from the Index of Relative Socioeconomic Advantage and Disadvantage Score (IRSAD Score) as the index of choice for this study. It is derived by principal components analysis of 21 separate variables such as low or high income, internet connection, unemployment, occupation and education. It does not include age or self-identification as of ATSI origin. The score is standardised against a mean of 1000 with a standard deviation of 100 with two thirds of SEIFA scores falling between 900 and 1100.

Remoteness of an area and relative access to infrastructure are also not included in the information used to construct SEIFA codes [16]. We have therefore also linked each candidate’s postcode to the Accessibility/Remoteness Index of Australia (ARIA) [18]. ARIA calculates remoteness as accessibility to some 201 service centres based on road distances. Remoteness values for 11,340 populated localities are derived from the road distance to service centres in four categories. Remoteness values for each populated locality are then interpolated to a 1 km grid that covers the whole of Australia and averages calculated for larger areas. ARIA values are grouped into one of five categories within the 0 – 12 continuous variable: Highly Accessible (ARIA score 0–1.84) - relatively unrestricted accessibility to a wide range of goods and services and opportunities for social interaction, Accessible (ARIA score >1.84 - 3.51) - some restrictions to accessibility of some goods, services and opportunities for social interaction, Moderately Accessible (ARIA score >3.51 -5.80) - significantly restricted accessibility of goods, services and opportunities for social interaction. Remote (ARIA score >5.80 - 9.08) - very restricted accessibility of goods, services and opportunities for social interaction, and Very Remote (ARIA score >9.08 - 12) - very little accessibility of goods, services and opportunities for social interaction.

Even though the total UMAT score alone is usually used in the ranking process at medical schools in Australia, each of the three component scores, UMAT-1 (Logical reasoning and problem solving), UMAT-2 (Understanding people) and UMAT-3 (Non-verbal reasoning) have different and independent constructs [19] and have therefore been independently evaluated in this study together with the total score. They are presented as percentile values to provide a more meaningful understanding of the relative magnitude of the associations of each score with predictor socio-demographic variables.

The project has been approved by the Human Research Ethics Committee at the University of Western Australia (file reference RA/4/1/2178).

### Statistics

Univariate comparisons of each demographic characteristic or each selection criteria utilised either independent sample T-tests, one-way analysis of variance (with post-hoc comparisons by Bonferroni correction), or cross-tabulation with generation of the chi-squared statistic, as appropriate. Multivariate analyses utilised linear regression to assess the independent relationships of total UMAT, UMAT-1, UMAT-2 and UMAT-3 with age, gender, type of secondary school, language spoken at home, country of origin, IRSAD decile, ARIA accessibility index and self-identification as ATSI. Further adjustment of each linear regression model by inclusion of 11 dummy variables for each year the UMAT was sat resulted in minimal change in both the B regression coefficients for each of the socio-economic predictor variables and the total variance explained by each model and have therefore not been included in the final analyses. All analyses were carried out utilising IBM SPSS Statistics Version 20.0.

## Results

### Age

**Figure 1:**
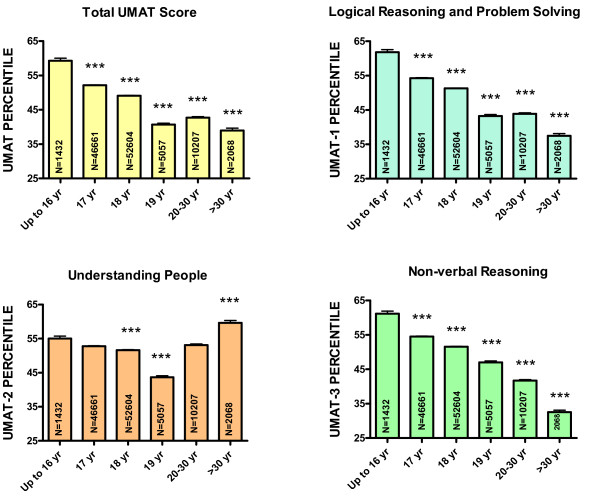
**Percentile score for total UMAT, UMAT-1, UMAT-2 and UMAT-3 by age for all Australian subjects first sitting the UMAT from 2000 to 2012 (N = 118,029).** (*** P < 0.001 – one way ANOVA post-hoc comparisons with Bonferroni correction).

Most Australians sitting the UMAT were school leavers with 84% of the sample 17 or 18 years of age. Performance in the UMAT decreased linearly with age (Figure 1) with those 30 yr and older predicted in multivariate linear regression (Table 1) to have a score 22.7 percentiles (95% CI 20.7,24.6) lower than those ≤ 16 yr in age (P < 0.001). This trend was present for UMAT-1 (27.2, 95% CI 25.3, 29.1, P < 0.001) (Table 2), not present for UMAT-2 (Table 3) and present for UMAT-3 (29.4, 95% CI 27.4, 31.4, P < 0.001) (Table 4). For UMAT-2, scores were significantly lower at all ages compared to those aged ≤ 16 yr (P < 0.001) except in those 30 yr and older where scores were not significantly different (Table 3).

**Table 1:** **Multivariate linear regression for total UMAT score (N = 109,880, r**^**2**^ **= 0.120)**

***Predictor variable (reference group in brackets)***	**B Coefficient**	**95% CI for B**	**Beta**	**P-Value**
***Age*** *(≤ 16 yr yr of age)*				
*17 yr*	−8.7	−10.2, -7.2	−0.147	<0.001
*18 yr*	−12.0	−13.5, -10.5	−0.207	<0.001
*19 yr*	−19.4	−21.0, -17.7	−0.133	<0.001
*20 - 30 yr*	−19.3	−20.9, -17.7	−0.183	<0.001
*> 30 yr*	−22.7	−24.6, -20.7	−0.100	<0.001
***Gender*** *(Females)*				
*Males*	3.6	3.3, 3.9	0.062	<0.001
***Language spoken at home*** *(English)*				
*Asian languages*	−10.4	−10.8, -10.0	−0.159	<0.001
*European languages*	−12.7	−13.8, -11.6	−0.063	<0.001
*Other languages*	−18.4	−19.6, -17.2	−0.083	<0.001
***School Type*** *(Government)*				
*Catholic*	−5.0	−5.5, -4.6	−0.068	<0.001
*Independent*	3.9	3.5, 4.3	0.064	<0.001
*Other*	−10.7	−11.9, -9.4	−0.049	<0.001
*TAFE*	−13.3	−15.7, -10.8	−0.030	<0.001
***State or territory*** *(NSW)*				
*ACT*	3.1	1.7, 4.5	0.013	<0.001
*NT*	−12.1	−14.4, -9.8	−0.031	<0.001
*QLD*	−5.9	−6.5, -5.3	−0.065	<0.001
*SA*	−9.0	−9.6, -8.3	−0.089	<0.001
*TAS*	1.3	0.2, 2.5	0.007	0.022
*VIC*	−6.6	−7.0, -6.2	−0.110	<0.001
*WA*	−5.9	−6.5, -5.3	−0.062	<0.001
***IRSAD Decile*** *(Deciles 1 and 2)*				
*Deciles 3 and 4*	3.9	3.2, 4.9	0.035	<0.001
*Deciles 5 and 6*	4.5	3.9, 5.4	0.053	<0.001
*Deciles 7 and 8*	7.1	6.6, 8.0	0.102	<0.001
*Deciles 9 and 10*	13.0	12.4, 13.8	0.224	<0.001
***ARIA Accessibility code*** *(Highly accessible)*				
*Accessible*	3.0	2.2, 3.8	0.022	<0.001
*Moderately accessible*	1.9	0.4, 3.4	0.007	0.015
*Remote*	1.9	−1.4, 3.9	0.004	0.161
*Very remote*	−1.5	−6.8, 4.7	−0.001	0.614
***Aboriginal & Torres Strait Islander*** *(non-ATSI)*				
*ATSI*	−9.6	−11.9, -7.3	−0.023	<0.001

**Table 2:** **Multivariate linear regression for UMAT-1 score (Logical reasoning and problem solving) (N = 109,880, r**^**2**^ **= 0.135)**

***Predictor variable (reference group in brackets)***	**B Coefficient**	**95% CI for B**	**Beta**	**P-Value**
***Age*** *(≤ 16 yr yr of age)*				
* 17 yr*	−9.2	−10.7, -7.7	−0.156	<0.001
* 18 yr*	−12.5	−14.0, -11.1	−0.216	<0.001
* 19 yr*	−18.9	−20.5, -17.2	−0.130	<0.001
* 20 - 30 yr*	−20.3	−21.9, -18.7	−0.192	<0.001
* > 30 yr*	−27.2	−29.1, -25.3	−0.120	<0.001
***Gender*** *(Females)*				
* Males*	7.0	6.7, 7.3	0.119	<0.001
***Language spoken at home*** *(English)*				
* Asian languages*	−12.5	−12.9, -12.1	−0.192	<0.001
* European languages*	−12.9	−14.0, -11.8	−0.064	<0.001
* Other languages*	−21.2	−22.4, -19.9	−0.096	<0.001
***School*** *(Government)*				
* Catholic*	−4.5	−4.9, -4.0	−0.061	<0.001
* Independent*	3.6	3.2, 4.0	0.060	<0.001
* Other*	−9.4	−10.6, -8.1	−0.043	<0.001
* TAFE*	−12.7	−15.2, -10.3	−0.029	<0.001
***State or territory*** *(NSW)*				
* ACT*	2.6	1.2, 3.4	0.011	<0.001
* NT*	−12.7	−15.0, -10.5	−0.033	<0.001
* QLD*	−5.4	−6.0, -4.8	−0.059	<0.001
* SA*	−9.8	−10.4, -9.2	−0.098	<0.001
* TAS*	1.3	0.1, 2.4	0.006	0.028
* VIC*	−5.0	−5.4, -4.6	−0.082	<0.001
* WA*	−4.2	−4.8, -3.6	−0.044	<0.001
***IRSAD Decile*** *(Deciles 1 and 2)*				
* Deciles 3 and 4*	3.9	3.1, 4.8	0.035	<0.001
* Deciles 5 and 6*	4.3	3.5, 5.1	0.051	<0.001
* Deciles 7 and 8*	6.6	5.9, 7.3	0.094	<0.001
* Deciles 9 and 10*	11.5	10.8, 12.2	0.199	<0.001
***ARIA Accessibility code*** *(Highly accessible)*				
* Accessible*	4.2	3.4, 5.0	0.031	<0.001
* Moderately accessible*	3.3	1.8, 4.8	0.013	<0.001
* Remote*	2.7	0.1, 5.3	0.006	0.040
* Very remote*	0.4	−5.2, 6.0	0.000	0.888
***Aboriginal & Torres Strait Islander*** *(non-ATSI)*				
* ATSI*	−8.9	−11.2, -6.7	−0.022	<0.001

**Table 3:** **Multivariate linear regression for UMAT-2 score (Understanding people) (N = 109,880, r**^**2**^ **= 0.113)**

***Predictor variable (reference group in brackets)***	**B Coefficient**	**95% CI for B**	**Beta**	**P-Value**
***Age*** *(≤ 16 yr yr of age)*				
*17 yr*	−3.8	−5.3, -2.3	−0.064	<0.001
*18 yr*	−5.1	−6.6, -3.6	−0.087	<0.001
*19 yr*	−11.1	−12.7, -9.4	−0.076	<0.001
*20 - 30 yr*	−4.7	−6.3, -3.1	−0.044	<0.001
*> 30 yr*	1.5	−0.5, 3.4	0.006	0.143
***Gender*** *(Females)*				
*Males*	−7.3	−7.6, -7.1	−0.125	<0.001
***Language spoken at home*** *(English)*				
*Asian languages*	−15.6	−15.9, -15.2	−0.238	<0.001
*European languages*	−9.7	−10.8, -8.5	−0.048	<0.001
*Other languages*	−14.9	−16.1, -13.7	−0.067	<0.001
***School*** *(Government)*				
*Catholic*	−1.3	−1.8, -0.9	−0.018	<0.001
*Independent*	3.4	3.0, 3.8	0.056	<0.001
*Other*	−10.1	−11.3, -8.8	−0.046	<0.001
*TAFE*	−10.7	−13.2, -8.2	−0.025	<0.001
***State or territory*** *(NSW)*				
*ACT*	1.9	0.5, 3.2	0.008	0.008
*NT*	−8.6	−10.9, -6.2	−0.022	<0.001
*QLD*	−4.6	−5.2, -4.0	−0.051	<0.001
*ISA*	−6.3	−6.9, -5.7	−0.063	<0.001
*TAS*	1.4	0.3, 2.6	0.007	0.014
*IVIC*	−5.7	−6.1, -5.3	−0.094	<0.001
*WA*	−5.5	−6.1, -4.9	−0.057	<0.001
***IRSAD Decile*** *(Deciles 1 and 2)*				
*Deciles 3 and 4*	2.9	2.1, 3.8	0.026	<0.001
*Deciles 5 and 6*	3.6	2.8, 4.4	0.042	<0.001
*Deciles 7 and 8*	5.4	4.7, 6.1	0.077	<0.001
*Deciles 9 and 10*	8.1	7.4, 8.8	0.140	<0.001
***ARIA Accessibility code*** *(Highly accessible)*				
*Accessible*	3.7	2.8, 4.5	0.027	<0.001
*Moderately accessible*	2.6	1.0, 4.1	0.010	0.001
*Remote*	4.4	1.8, 7.1	0.010	0.001
*Very Remote*	1.4	−4.3, 7.0	0.001	0.635
***Aboriginal & Torres Strait Islander*** *(non-ATSI)*				
*ATSI*	−6.2	−8.5, -3.9	−0.015	<0.001

**Table 4:** **Multivariate linear regression for UMAT-3 score (Non-verbal reasoning) (N = 109,880, r**^**2**^ **= 0.098)**

***Predictor variable (reference group in brackets)***	**B Coefficient**	**95% CI for B**	**Beta**	**P-Value**
***Age*** *(≤ 16 yr yr of age)*				
*17 yr*	−7.2	−8.8, -5.7	−0.122	<0.001
*18 yr*	−10.4	−11.9, -8.8	−0.178	<0.001
*19 yr*	−15.6	−17.3, -13.9	−0.107	<0.001
*20 - 30 yr*	−21.0	−22.6, -19.4	−0.198	<0.001
*> 30 yr*	−29.4	−31.4, -27.4	−0.130	<0.001
***Gender*** *(Females)*				
*Males*	8.1	7.8, 8.4	0.138	<0.001
***Language spoken at home*** *(English)*				
*Asian languages*	2.4	2.0, 2.8	0.037	<0.001
*European languages*	−7.2	−8.3, -6.0	−0.036	<0.001
*Other languages*	−8.9	−10.1, -7.6	−0.040	<0.001
***School*** *(Government)*				
*Catholic*	−5.4	−5.8, -4.9	−0.073	<0.001
*Independent*	2.4	2.0, 2.8	0.039	<0.001
*Other*	−5.9	−7.2, -4.7	−0.027	<0.001
*TAFE*	−9.1	−11.6, -6.6	−0.021	<0.001
***State or territory*** *(NSW)*				
*ACT*	2.0	0.6, 3.3	0.008	0.005
*NT*	−7.5	−10.1, -5.4	−0.020	<0.001
*QLD*	−4.0	−4.6, -3.4	−0.044	<0.001
*SA*	−5.0	−5.6, -4.4	−0.050	<0.001
*TAS*	−0.04	−1.1, 1.2	0.000	0.945
*VIC*	−4.8	−5.2, -4.4	−0.080	<0.001
*WA*	−3.9	−4.6, -3.3	−0.041	<0.001
***IRSAD Decile*** *(Deciles 1 and 2)*				
*Deciles 3 and 4*	2.7	1.9, 3.7	0.025	<0.001
*Deciles 5 and 6*	3.0	2.4, 3.9	0.037	<0.001
*Deciles 7 and 8*	5.5	4.8, 6.3	0.079	<0.001
*Deciles 9 and 10*	11.6	11.0, 12.4	0.202	<0.001
***ARIA Accessibility code*** *(Highly accessible)*				
*Accessible*	−0.08	−0.9, 0.8	0.000	0.852
*Moderately accessible*	−1.0	−2.6, 0.5	−0.004	0.182
*Remote*	−2.5	−5.5, 0.2	−0.006	0.068
*Very remote*	−4.1	−9.8, 1.6	−0.003	0.163
***Aboriginal & Torres Strait Islander*** *(non-ATSI)*				
*ATSI*	−7.7	−10.1, -5.4	−0.019	<0.001

### Gender

**Figure 2:**
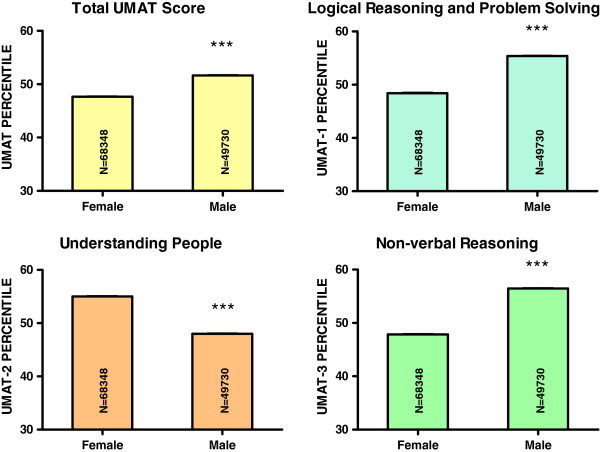
**Percentile score for total UMAT, UMAT-1, UMAT-2 and UMAT-3 by gender for all Australian subjects first sitting the UMAT from 2000 to 2012 (N = 118,078).** (*** P < 0.001 – one way ANOVA post-hoc comparisons with Bonferroni correction).

Females comprised 58% of the sample. Males performed better for total UMAT score by 3.6 percentiles (95% CI 3.3, 3.9, P < 0.001), UMAT-1 by 7.0 percentiles (95% CI 6.7, 7.3, P < 0.001) and UMAT-3 by 8.1 percentiles (95% CI 7.8, 8.4, P < 0.001), but worse than females in UMAT-2 with scores lower by 7.3 percentiles (95% CI 7.1, 7.6, P < 0.001) (Figure 2) (Tables 1, 2, 4).

### Language at home

**Figure 3:**
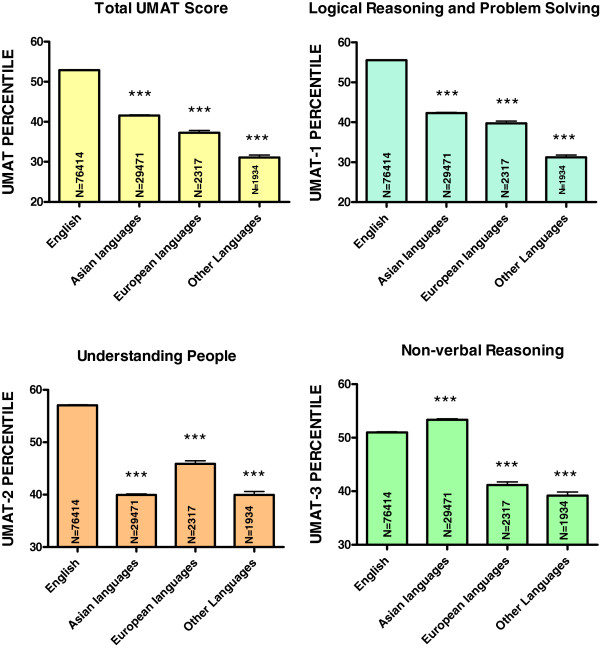
**Percentile score for total UMAT, UMAT-1, UMAT-2 and UMAT-3 by language spoken at home for all Australian subjects first sitting the UMAT from 2000 to 2012 (total number of applicants N = 110,136).** (*** P < 0.001 – one way ANOVA post-hoc comparisons with Bonferroni correction).

When compared to those who only spoke English at home (69.4% of the sample), scores for total UMAT were lower by 10.4 percentiles (95% CI 10.0, 10.8, P < 0.001) for those speaking Asian languages (26.7% of the sample), lower by 12.7 percentiles (95% CI 11.6, 13.8, P < 0.001) for those speaking European languages (2.1% of the sample) and lower by 18.4 percentiles (95% CI 17.2, 19.6, P < 0.001) for those speaking any other language (1.8% of the sample) (Table 1) (Figure 3). Similar differentials were evident for UMAT-1 and UMAT-2 for anyone speaking a language other than English at home (Tables 2, 3) (Figure 3). However for UMAT-3, for those speaking Asian languages the score was higher by 2.4 percentiles (95% CI 2.0, 2.8, P < 0.001) but remained lower for those speaking European languages by 7.2 percentiles (95% CI 6.0, 8.3, P < 0.001) and by 8.9 percentiles (95% CI 7.6, 10.1, P < 0.001) for those speaking any other language (Table 4) (Figure 3).

### Secondary school type

**Figure 4:**
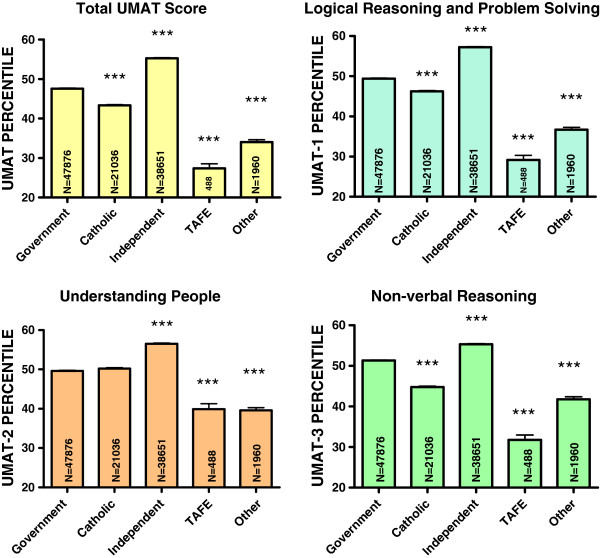
**Percentile score for total UMAT, UMAT-1, UMAT-2 and UMAT-3 by type of secondary school for all Australian subjects first sitting the UMAT from 2000 to 2012 (total number of applicants N = 110,011).** (*** P < 0.001 – one way ANOVA post-hoc comparisons with Bonferroni correction).

The largest proportion of students were from predominantly a government secondary school background (43.6%) followed by independent secondary schools (35.1%), catholic secondary schools (19.1%) and other secondary school or TAFE (2.2%). Students attending government schools scored lower for total UMAT by 3.9 percentiles (95% CI 3.5, 4.3) compared to those from independent schools (P < 0.001). Those attending government schools scored higher than those attending Catholic schools (by 5.0 percentiles, 95% CI 4.6, 5.5) (P < 0.001), TAFE colleges (13.3 percentiles, 95% CI 10.8, 15.7) (P < 0.001) and all Other institutions (10.7 percentiles, 95% CI 9.4, 11.9) (P < 0.001) (Table 1, Figure 4). Nearly identical results were seen for each section of the UMAT (Tables 2, 3, 4).

### Accessibility/remoteness index of Australia

**Figure 5:**
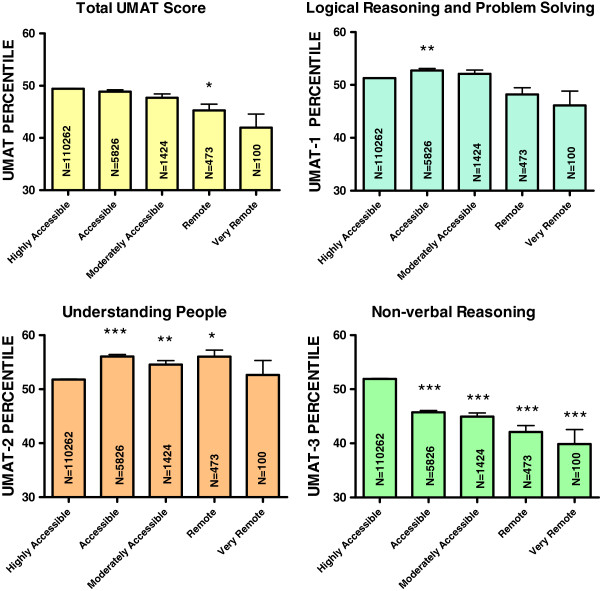
**Percentile score for total UMAT, UMAT-1, UMAT-2 and UMAT-3 by accessibility/remoteness index of Australia for all Australian subjects first sitting the UMAT from 2000 to 2012 (N = 118,085).** (* P < 0.05, ** P < 0.01, *** P < 0.001 – one way ANOVA post-hoc comparisons with Bonferroni correction).

Students from areas defined by ARIA score as highly accessible comprised 93.4% of the sample. Those from accessible areas comprised 4.9%, moderately accessible 1.2% and remote or very remote only 0.5%. The associations with ARIA score were inconsistent across the 3 sections of UMAT. Compared to highly accessible areas, UMAT-1 scores were higher in those from areas that were defined as accessible (4.2 percentiles, 95% CI 3.4, 5.0) (P < 0.001) or moderately accessible (3.3 percentiles, 95% CI 1.8, 4.8) (P < 0.001) (Table 2, Figure 5). UMAT-2 scores were higher in those from areas defined as accessible (3.7 percentiles, 95% CI 2.8, 4.5) (P < 0.001), moderately accessible (2.6 percentiles, 95% CI 1.0, 4.1) (P = 0.001) or remote (4.4 percentiles, 95% CI 1.8, 7.1) (P = 0.001) (Table 3, Figure 5). In contrast, compared to highly accessible areas, UMAT-3 scores were progressively lower in the univariate analysis (Figure 5) with this trend no longer statistical significance in the multivariate analysis (Table 4). These divergent associations overall resulted in the multivariate analysis predicting total UMAT scores which were higher in those from areas that were defined as accessible (3.0 percentiles, 95% CI 2.2, 3.8) (P < 0.001) or moderately accessible (1.9 percentiles, 95% CI 0.4, 3.4) (P = 0.015) compared to those from highly accessible areas (Table 1).

### Aboriginal or Torres Strait Islander

Only a relatively small number of those self-identifying as Aboriginal or Torres Strait Islander (ATSI) (N = 556, 0.5%) sat the UMAT during the time period. Their scores on total UMAT were lower by 9.6 percentiles (95% CI 7.3, 11.9, P < 0.001), UMAT-1 by 8.9 percentiles (95% CI 6.7, 11.2, P < 0.001) and UMAT-2 by 6.2 percentiles (95% CI 3.9, 8.5, P < 0.001) and UMAT-3 by 7.7 percentiles (95% CI 5.4, 10.1, P < 0.001) (Tables 1, 2, 3, 4).

### Socio-economic advantage and disadvantage

**Figure 6:**
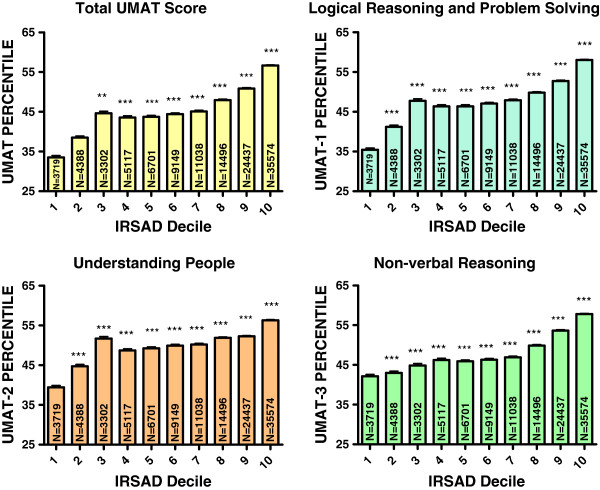
**Percentile score for total UMAT, UMAT-1, UMAT-2 and UMAT-3 by deciles for index of relative socio-economic advantage and disadvantage score for all Australian subjects first sitting the UMAT from 2000 to 2012 (N = 117,347).** (** P < 0.01, *** P < 0.001 – one way ANOVA post-hoc comparisons with Bonferroni correction).

Australians sitting the UMAT largely came from the top 2 deciles for IRSAD score (50.8%) with only 6.9% from the bottom 2 deciles. For every UMAT section, scores diminished progressively with increasing socio-economic disadvantage and decreasing socio-economic advantage. This translated into IRSAD decile being the strongest predictor of total UMAT score in the final multivariate model. It resulted in total UMAT scores in those in the highest 2 deciles that were 13.0 percentiles higher (95% CI, 12.4, 13.8) (P < 0.001) than those achieved by candidates from the lowest 2 deciles (Table 1, Figure 6). In subsequent analyses the magnitude and profile of this relationship remained similar for each language group (data not shown).

Many of the predictor variables were also related to the IRSAD decile of origin of each candidate. Approximately 49% females were in the top 2 deciles compared to 53% of males (χ^2^ = 173, P < 0.001). With increasing age there was a progressively diminishing proportion in the top 2 deciles (from 53% of 17 year-olds down to 46% of those >30 yr, χ^2^ = 126, P < 0.001). For those who attended independent secondary schools, 63% were from the highest 2 IRSAD deciles compared to 46% from government schools and 39% from Catholic schools (χ^2^ = 4170, P < 0.001). Approximately 54% of those in the most highly accessible areas were in the top 2 deciles compared to only 3 to 10% of those in any area with higher ARIA scores (χ^2^ = 7653, P < 0.001). Approximately 53% of those where English was the language spoken at home were in the top 2 deciles compared to 46% of those speaking Asian languages, 42% of those speaking European languages and 37% of those speaking any other language (χ^2^ = 703, P < 0.001). Of those identifying as ATSI, 32% were within the highest 2 IRSAD deciles compared to 51% of non-ATSI (χ^2^ = 82.7, P < 0.001).

### State or territory of origin

Nearly two thirds of the cohort (64.7%) were from Australia’s 2 most populous states, New South Wales and Victoria. With New South Wales as the comparator state, higher mean scores were seen in candidates from the Australian capital Territory (ACT) and Tasmania (TAS) while lower mean scores were seen in Victoria (VIC), Queensland (QLD), South Australia (SA), Western Australia (WA) and the Northern Territory (NT) (Tables 1, 2, 3, 4).

Many of these state-based differences were confounded by substantial socio-demographic differences across the states. For example TAS had the largest proportion of subjects speaking English at home (93% of all TAS candidates) while those with an Asian language background were heavily concentrated in NSW and VIC (38% and 35% of all NSW and VIC candidates respectfully) (χ^2^ = 2338, P < 0.001). When broken down by state, 99.8% of those from the ACT were within the top 2 IRSAD deciles compared to 63% of those from WA, 54% of those from QLD, 51% of those from VIC, 50% of those from NSW, 35% of those from SA, 28% of those from TAS and 17% of those from the NT (χ^2^ = 4402, P < 0.001). The relative profile of government vs independent vs catholic school education also varied significantly across states as did the relative proportion of candidates from a rural background. Finally the age first sitting the UMAT was different across states with more 17 yo candidates from WA and QLD and more 18 yo candidates from VIC, TAS and the ACT.

## Discussion

We have observed a consistent relationship between a number of socioeconomic indices and performance over more than a decade by Australian candidates in the UMAT. The UMAT total score and performance in each of its subsections was linked to an index of relative socio-economic advantage and disadvantage generated from the postcode of the correspondence address at the time of sitting the UMAT. Being from the top 2 socio-economic deciles as determined by a broad spectrum of indices generated from Australian census data was positively associated with UMAT performance. In addition, it was positively associated with prior secondary education in a fee-paying independent school and negatively associated with self-identification as being of ATSI origin. These results raise the prospect of a diversity limiting effect of selection processes at universities that utilise the UMAT.

A recent Canadian study of applicants to 6 medical schools also utilised a measure of socio-economic status linked by postcode to community size and income levels [12]. They identified an association between lower performance in the MCAT in those from smaller communities but saw no relationship with income levels. Academic performance as measured by GPA was linked to income levels but not to community size while interview scores were unrelated to either of these socio-economic measures. Similar to our finding, they also reported lower MCAT scores in applicants of self-declared aboriginal origin. Although this was interpreted as another indication that performance in aptitude tests might be influenced by socio-economic status, the authors also allowed that a contributing factor may have been the widespread availability of facilitative admissions processes for indigenous Canadians which could have created a larger pool of candidates applying with generally lower admission test scores [20].

A study of all UK candidates who sat the inaugural UKCAT test in 2006 [13] is probably more relevant in comparing our observations of a potential socio-economic influence on UMAT performance. It similarly is administered predominantly to secondary school leaver applicants to medical and dental schools and comprises 4 sections with some similarities to the UMAT – verbal reasoning, quantitative reasoning, abstract reasoning and decision analysis [13]. They used 3 potential indicators of socioeconomic status – ethnicity, parental occupation and education at independent/grammar schools – and found that male sex, white ethnicity, having parents from a professional/managerial background and independent or grammar schooling were each independent indicators of more favourable UKCAT performance. This test like the UMAT had been introduced in the expectation of increasing diversity and fairness in selection but the authors have concluded on the basis of this data that a significant socio-economic influence on test results may still remain.

The more important outcome of course, is whether such an association between test performance and socio-economic background ultimately translates into an actual impact on medical school selection. In this regard a follow-up study [4] has assessed the impact of use of the UKCAT in the 2009 cohort on subsequent selection into medical school. If it was utilised as a weighted factor in selection or as a tie-breaker for borderline applicants, those candidates from the lowest socioeconomic background were approximately 30-50% less likely to be given a conditional or unconditional offer of a place at medical school. However, if it was used as a threshold score to decide whether or not a candidate was to receive an interview, socio-economic background was not a significant predictor of whether an offer would be made. This translated into medical schools that used the UKCAT as a threshold being 3.6 times more likely to offer a place to someone from a low socioeconomic background than schools that used it as a weighted factor or as a borderline tie-breaker. The corollary however, was that where offers were made to students conditional on a certain level of academic achievement, conversion from a conditional to an unconditional offer for those of low socio-economic status was nearly 60% less likely in applicants to schools that used the UKCAT as a threshold parameter for selection. While this study was able to compare these different approaches across 22 medical schools that utilised the UKCAT in selection, it was not able to compare the relative socio-demographic makeup of selected students in schools not using the UKCAT. It also did not report on the actual change in the socio-demographic make-up of participating medical schools before and after incorporation of the UKCAT into selection processes [4].

The UMAT has been correlated, albeit weakly, with academic performance [5,14,15]. Given that academic performance is a consistently utilised selection factor for medical schools globally, it is likely that UMAT attracts a cohort of students who are academically performing at the higher end of the spectrum. Better academic performance by school leavers has been repeatedly linked to increased socio-economic advantage with academic achievement highest at entry into medical school in those from less materially disadvantaged households [20]. The association of UMAT scores with IRSAD decile therefore may at least in part be dictated by a stronger academic performance in those who choose to sit the UMAT. In this cohort we did not have any data on candidate’s prior academic performance, the UMAT for the most part being sat in the final year of secondary school before tertiary entrance academic results are known. However, we have been able to investigate this hypothesis in a cohort of students who have entered our medical school from secondary school over a 12 year period using a combination of UMAT, academic performance and a score from a structured interview [5]. At least in part the hypothesis was supported in this much smaller and highly selected cohort by the finding of a substantial attenuation of the relationship between UMAT score and IRSAD decile when prior academic performance was taken into account, with parameter estimates for the magnitude of the association reducing by 30 to 50 percent (Puddey IB – personal communication).

In the UK, ethnicity has been recognized as a potential confounding factor when attempting to unpick the relationship between socio-economic background and access into medical schools [21]. We were unable to assess ethnicity but by using language spoken at home as a surrogate, we found that approximately 27% of those who sat the UMAT from 2000 to 2012 came from Asian language backgrounds. We have previously reported an over-representation of Asian students applying to and being admitted into medical schools in Australia relative to background prevalence in the population [5]. A similar phenomenon has been observed in both the UK [22] and in New Zealand [23]. In our cohort, fewer Asian language students were in the top 2 IRSAD deciles compared to English language students and their overall UMAT performance was generally weaker. To an extent therefore, ethnicity may have been an additional factor in the pathway linking socio-economic background to performance in the UMAT. However, when we studied each major language group separately, the magnitude and pattern of the relationship between IRSAD decile and UMAT performance was almost identical.

It is recognised that a high percentage of candidates for aptitude tests for medical school entry undergo some prior commercial coaching in an attempt to optimise performance. In an Australian context this has been reported to be as high as 56% [14] while for the MCAT a review identified 4 studies in the area where the prevalence of coaching varied between 22%, 25%, 38% and 72% of candidates respectively [24]. Given this high prevalence and that UMAT coaching courses are relatively expensive it is possible that prior coaching could have confounded our results. More students from higher socio-economic backgrounds may have had access to coaching which enhanced their subsequent UMAT performance. Also UMAT coaching and practice may be more systematic and organised within fee-paying independent schools [14]. Whether this has influenced our results remains controversial, however, with recent reports indicating either no effect of preparation courses on UMAT performance or only a weak effect in improving Section 3 - non-verbal reasoning in selected students [12,25].

The findings of significant differences in performance by Australian state or territory of abode were a somewhat surprising finding but look likely to be linked to considerable differences in socio-demographic profiles across the states. The top performing states were those with the highest proportion of students in the top 2 IRSAD deciles. Age of students at entry to and exit from primary and secondary schooling varies across states and would have contributed to a changing age profile across states for age first sitting the UMAT. Relative proportions of students from Asian language versus English language backgrounds were markedly dissimilar across states and there were differences in the proportions of those from rural backgrounds. Finally, the mix of those receiving government vs independent vs Catholic school education also differed significantly by state.

Our estimates of the relative contribution that socio-demographic factors make to the overall variance in UMAT scores were substantially higher than those previously identified in the 2012 ACER report on the UMAT [1] with estimates from our linear regression models of 12% for total UMAT score, 13.5% for UMAT-1, 11.3% for UMAT2 and 9.8% for UMAT-3. This represents a nearly 3-fold higher estimate for the total UMAT score and relates to both the broader range of variables included in our analysis as well as the increase in power afforded by including all those who sat for the test over a 13 year period.

### Study limitations

The study is cross sectional in nature and hence a causal link between socio-economic background and UMAT performance has not been established by these data. UMAT score statistics (mean and standard deviation) have varied over the years, however large numbers in the cohorts and the use of percentile ranks provide a measure independent of such statistics. The SEIFA codes are generated from a suite of summary measures in defined areas based on census information and do not apply to an individual person or dwelling. Using an individual’s postcode is therefore only a surrogate for true socio-economic status with SEIFA codes imputing an index based on the level of socio-economic disadvantage for all people living in a defined area. It is likely a significant proportion of candidates would have been living in student dormitories or lodgings near their university or secondary school rather than their usual place of residence and this may have weakened the true underlying strength of the associations we have reported. On the other hand aggregating 21 socioeconomic indicators into a single index and then further aggregating by postcode would reduce the variance associated with each indicator and may inflate the strength of the associations reported. Finally socio-economic status linked to an area is not static over time and we have used the 2006 SEIFA codes over a period that spans 2000 to 2012 again potentially weakening the relative accuracy of imputed socio-economic status.

## Conclusions

We have observed a direct relationship between socio-economic background and performance in the UMAT. This observation was consistent for all sections of the UMAT and across a number of socio-demographic variables including IRSAD decile, school background and self-identification as being of ATSI origin. The association was similar to that which has already been well documented for prior academic performance. Therefore in Australia, where UMAT is often utilised alone to select candidates for interview, there may be important implications of these observations for current selection processes that may otherwise be seeking to widen medical school access to those students from a broader socio-economic base through use of an aptitude test. A prospective study of the potential impact of the UMAT on the profile of students selected to Australian medical schools is clearly warranted. The quarantining of places through affirmative action pathways to admit students from lower socio-economic backgrounds who have reached acceptable threshold scores in each section of the UMAT may be a necessary complementary approach for ensuring student diversity.

## Competing interests

IP is the representative for the University of Western Australia on the UMAT Consortium Board of Management. AM is a member of the UMAT Test Management Committee and Chair of both the UMAT Technical Subcommittee and the UMAT Research Subcommittee.

## Authors’ contributions

IP contributed to the conception and design of the study, acquisition, analysis and interpretation of the data; and the initial drafting and final revision of the manuscript. AM contributed to the conception and design of the study, interpretation of the data; and final revision of the manuscript for important intellectual content. Both authors read and approved the final manuscript.

## Pre-publication history

The pre-publication history for this paper can be accessed here:

http://www.biomedcentral.com/1472-6920/13/155/prepub
